# Crystal structure of *trans*-di­fluoridotetra­kis(pyridine-κ*N*)chromium(III) tri­chlorido­(pyridine-κ*N*)zincate monohydrate from synchrotron data

**DOI:** 10.1107/S160053681402145X

**Published:** 2014-10-04

**Authors:** Dohyun Moon, Jong-Ha Choi

**Affiliations:** a Pohang Accelerator Laboratory, POSTECH, Pohang 790-784, Republic of Korea; b Department of Chemistry, Andong National University, Andong 760-749, Republic of Korea

**Keywords:** crystal structure, fluoride ligand, pyridine ligand, *trans*-isomer, chromium(III) complex

## Abstract

In the asymmetric unit of the title compound, [CrF_2_(C_5_H_5_N)_4_][ZnCl_3_(C_5_H_5_N)]·H_2_O, there are two independent complex cations, one tri­chlorido­(pyridine-κ*N*)zincate anion and one solvent water mol­ecule. The cations lie on inversion centers. The Cr^III^ ions are coordinated by four pyridine (py) N atoms in the equatorial plane and two F atoms in a *trans* axial arrangement, displaying a slightly distorted octa­hedral geometry. The Cr—N(py) bond lengths are in the range 2.0873 (14) to 2.0926 (17) Å while the Cr—F bond lengths are 1.8609 (10) and 1.8645 (10) Å. The [ZnCl_3_(C_5_H_5_N)]^−^ anion has a distorted tetra­hedral geometry. The Cl atoms of the anion were refined as disordered over two sets of sites in a 0.631 (9):0.369 (9) ratio. In the crystal, two anions and two water mol­ecules are linked *via* O—H⋯Cl hydrogen bonds, forming centrosymmetric aggregates. In addition, weak C—H⋯Cl, C—H⋯π and π–π stacking inter­actions [centroid–centroid distances = 3.712 (2) and 3.780 (2)Å] link the components of the structure into a three-dimensional network.

## Abstract 2

The Cr^III^ atoms in the title compound show a distorted octa­hedral coordination with four pyridine N atoms in the equatorial plane and two F atoms in axial positions. The [ZnCl_3_(C_5_H_5_N)]^−^ anion has a distorted tetra­hedral geometry.

## Chemical context   

Anionic species play very important roles in chemistry, medicine, catalysis, mol­ecular assembly, biology and environmental processes, yet their binding characteristics have not received much recognition (Martínez-Máñez & Sancenón, 2003 ▶; Fabbrizzi & Poggi, 2013 ▶). The study of the effect of anions and geometric isomers in octa­hedral metal complexes may be expected to yield a great variety of new structures and properties of both chemical and biological significance. Octa­hedral Cr^III^ complexes and their 3*d*–4*f* clusters containing lanthanides revealing paramagnetic features are of great importance for the development of new mol­ecule-based magnets and solid-state laser materials (Powell, 1998 ▶; Dreiser *et al.*, 2012 ▶; Singh *et al.*, 2013 ▶). We are therefore inter­ested in the preparation, crystal structures and spectroscopic properties of chromium(III) complexes containing mixed various ligands (Choi, 2000*a* ▶,*b* ▶; Choi *et al.*, 2004 ▶, 2006 ▶; Choi & Moon, 2014 ▶).
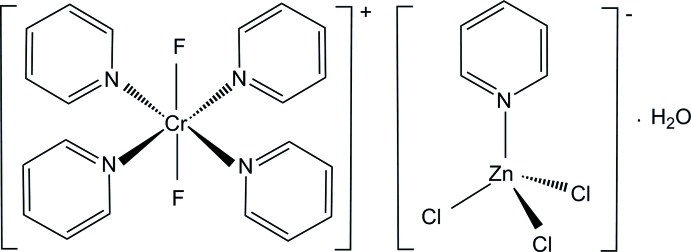


 Here we report the structure of [CrF_2_(py)_4_][ZnCl_3_(py)]·H_2_O, where py is the pyridine (C_5_H_5_N), in order to establish the exact arrangement of four py mol­ecules, two F atoms, counter-anion and water mol­ecule. This is another example of a *trans*-[CrF_2_(py)_4_]^+^ structure but with a different counter-anion system (Fochi *et al.*, 1991 ▶; Moon & Choi, 2013 ▶; Moon *et al.*, 2014 ▶; Singh *et al.*, 2013 ▶).

## Structural commentary   

**Figure 1:**
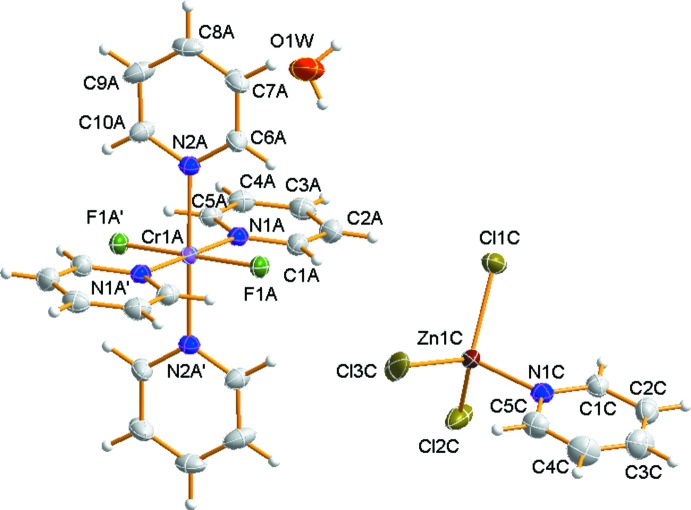
The mol­ecular structure of the title compound showing 50% probability displacement ellipsoids. Only one of the independent cations is shown. The minor disorder component of the anion is not shown. The primed atoms are related by the symmetry code (−*x*, −*y* + 1, −*z*).

In the mol­ecular structure, there are two independent Cr^III^ complex cations in which the four nitro­gen atoms of four py ligands occupy the equatorial sites and the two F atoms coordinate to the Cr atom in a *trans* configuration. An ellipsoid plot of one independent complex cation, the unique ZnCl_3_(py)^−^ anion and one water mol­ecule in the title compound is shown in Fig. 1 ▶.

The Cr—N(py) bond lengths range from 2.0873 (14) to 2.0926 (17) Å and the Cr—F bond lengths are 1.8609 (10) and 1.8645 (10) Å (Table 1 ▶). These Cr—N(py) and Cr—F bond lengths are in good agreement with those observed in *trans*-[CrF_2_(py)_4_]PF_6_, *trans-*[CrF_2_(py)_4_]ClO_4_, *trans-*[CrF_2_(py)_4_]_2_NaClO_4_ and *trans*-[CrF_2_(py)_4_][Cr(py)_4_F(μ-F)Li(H_2_O)_3_][Cr(py)_4_F(μ-F)Li(H_2_O)_4_]Cl_5_·6H_2_O (Fochi *et al.*, 1991 ▶; Moon & Choi, 2013 ▶; Moon *et al.*, 2014 ▶; Birk *et al.*, 2010 ▶). The Cr—F bond lengths are also similar to the values found in *trans-*[Cr(15aneN_4_)F_2_]ClO_4_ (15aneN_4_ = 1,4,8,12-tetraaza­cyclo­penta­decane) and *trans-*[Cr(2,2,3-tet)F_2_]ClO_4_ (2,2,3-tet = 1,4,7,11-tetraazaundecane) (Choi *et al.*, 2006 ▶; Choi & Moon, 2014 ▶). However, the Cr—F bond lengths are somewhat shorter than those found for bridging fluorides [1.9045 (14)–1.9145 (14) Å; Dreiser *et al.*, 2012 ▶).

**Figure 2:**
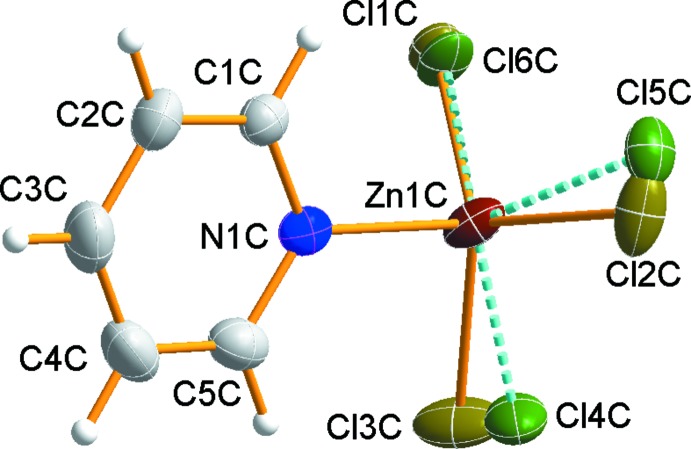
The mol­ecular structure of the anion. The minor disorder component is shown with dashed lines.

The [ZnCl_3_(py)]^−^ anion and uncoordinating water mol­ecule remain outside the coordination sphere. In the counter-anion, the Zn^II^ ion is in a distorted tetra­hedral environment, coordinated by one N atom of the py ligand and by three Cl atoms. The Cl atoms of the anion were refined as disordered over two sets of sites in a 0.631 (9):0.369 (9) ratio (Fig. 2 ▶). The Zn—Cl distances, ranging from 2.126 (14) to 2.360 (2) Å, and the Zn—N(py) distance of 2.075 (2) Å are in agreement with those found in the anion of [Cr(acacen)(py)_2_][ZnCl_3_(py)] [acacen = *N*,*N*′-ethylenebis(acetylacetoneiminato)] (Toscano *et al.*, 1994 ▶). The mean Cl—Zn—Cl angle of 115.22° is larger than the corresponding tetra­hedral angle and the mean Cl—Zn—N angle of 105.45 (10)°. The charge of the tri­chlorido­(pyridine)­zincate anion is counter-balanced by two half *trans*-[CrF_2_(py)_4_]^+^ cations. The complex cations lie on inversion centers and therefore the cations have exact mol­ecular *C_i_* symmetry.

## Supra­molecular features   

**Figure 3:**
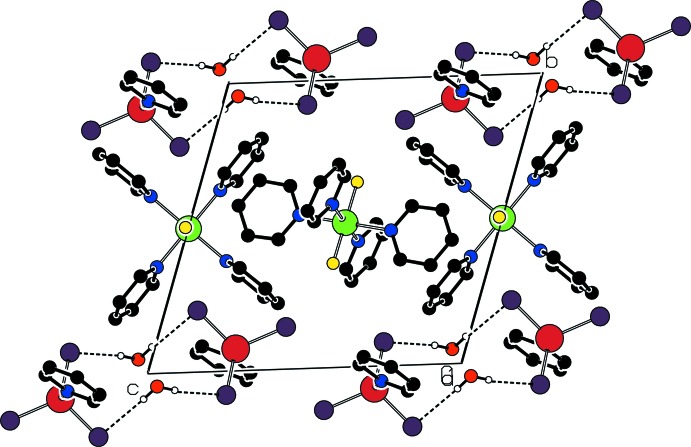
Part of the crystal structure with hydrogen bonds shown as dashed lines.

In the crystal, two anions and two water mol­ecules are linked *via* O—H⋯Cl hydrogen bonds, forming centrosymmetric aggregates with 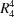
(12) rings (Fig. 3 ▶). In addition, weak C—H⋯Cl (Table 2 ▶), C—H⋯π (Table 3 ▶) and π–π stacking inter­actions link the components of the structure into a three-dimensional network. The centroid–centroid distances of the π–π stacking inter­actions are *Cg*1⋯*Cg*2(−1 + *x*, *y*, *z*) = 3.712 (2) and *Cg*3⋯*Cg*4 3.780 (2) Å, Where *Cg*1, *Cg*2, *Cg*3 and *Cg*4 are the centroids defined by ring atoms N1*A*/C1*A*–C5*A*, N1*C*/C1*C*–C5*C*, N2*B*/C6*B*–C10*B* and N2*A*/C6*A*–C10*A*, respectively.

## Synthesis and crystallization   

All chemicals were reagent grade materials and used without further purification. The starting material, *trans*-[CrF_2_(py)_4_]ClO_4_ was prepared according to the literature (Glerup *et al.*, 1970 ▶). The crude *trans*-[CrF_2_(py)_4_]ClO_4_ (0.2 g) was dissolved in 10 mL water. The 10 mL solution of 1*M* HCl and 0.5 g of ZnCl_2_ were added to this solution. The mixture was refluxed at 328 K for 30 min and then cooled to room temperature. The crystalline product which formed was filtered, washed with cold 2-propanol and diethyl ether. Recrystallization from a hot aqueous solution of the title compound yielded purple crystals suitable for X-ray structure analysis.

## Refinement   

Crystal data, data collection and structure refinement details are summarized in Table 4 ▶. C–bound H–atoms were placed in calculated positions (C—H = 0.95 Å) and were included in the refinement in the riding-model approximation with *U*_iso_(H) set to 1.2*U*_eq_(C). The hydrogen atoms of the solvent water mol­ecule were refined with *U*_iso_(H) set to 1.5 *U*_eq_(O) and geometrically restrained to O—H = 0.86 (1) and H⋯H 1.34 (2) Å. The Cl atoms of the anion were refined as disordered over two sets of sites with refined occupancies of 0.631 (9) and 0.369 (9), respectively.

## Supplementary Material

Crystal structure: contains datablock(s) I. DOI: 10.1107/S160053681402145X/lh5726sup1.cif

Structure factors: contains datablock(s) I. DOI: 10.1107/S160053681402145X/lh5726Isup2.hkl

CCDC reference: 1026562

Additional supporting information:  crystallographic information; 3D view; checkCIF report

## Figures and Tables

**Table 1:** Selected bond lengths ()

Cr1*A*F1*A*	1.8645(10)	Cr2*B*N1*B*	2.0916(15)
Cr1*A*N2*A*	2.0873(14)	Zn1*C*N1*C*	2.0752(16)
Cr1*A*N1*A*	2.0926(17)	Zn1*C*Cl2*C*	2.188(2)
Cr2*B*F1*B*	1.8609(10)	Zn1*C*Cl3*C*	2.302(2)
Cr2*B*N2*B*	2.0886(17)	Zn1*C*Cl1*C*	2.303(8)

**Table 2:** Hydrogen-bond geometry (, )

*D*H*A*	*D*H	H*A*	*D* *A*	*D*H*A*
O1*W*H1*O*1Cl2*C* ^i^	0.86(1)	2.41(2)	3.263(6)	173(8)
O1*W*H2*O*1Cl3*C* ^ii^	0.86(1)	2.48(4)	3.281(5)	157(8)
O1*W*H2*O*1Cl4*C* ^ii^	0.86(1)	2.22(2)	3.053(6)	165(8)
C2*B*H2*B*Cl3*C*	0.95	2.81	3.749(4)	170
C3*B*H3*B*Cl3*C* ^ii^	0.95	2.82	3.511(3)	130
C3*C*H3*C*Cl2*C* ^iii^	0.95	2.71	3.627(4)	162
C4*A*H4*A*Cl1*C* ^iv^	0.95	2.82	3.717(8)	158
C10*A*H10*A*Cl3*C* ^v^	0.95	2.86	3.617(4)	137
C10*B*H10*B*Cl1*C* ^vi^	0.95	2.73	3.534(9)	142

Symmetry codes: (i) 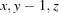; (ii) 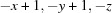; (iii) 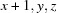; (iv) 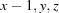; (v) 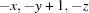; (vi) 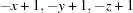.

**Table 3:** CH interaction geometry (,) *Cg*1*Cg*4 are the centroids defined by the ring atoms N2*A*/C6*A*C10*A*, N1*B*/C1*B*C5*B*, N1*A*/C1*A*C5*A* and N1*C*/C1*C*C5*C*, respectively.

DH*Cg*	DH	H*Cg*	D*Cg*	DH*Cg*
C4*C*H4*C* *Cg*1^i^	0.95	2.82	3.630(3)	144
C6*A*H6*A* *Cg*2	0.95	2.81	3.579(2)	139
C6*B*H6*B* *Cg*3	0.95	2.90	3.660(2)	138
C8*A*H8*A* *Cg*4^ii^	0.95	2.73	3.558(3)	147

Symmetry codes: (i) *x*+1, *y*+1, *z*; (ii) *x*1, *y*1, *z*.

**Table 4:** Experimental details

Crystal data	
Chemical formula	[CrF_2_(C_5_H_5_N)_4_][ZnCl_3_(C_5_H_5_N)]H_2_O
*M*_r_	675.23
Crystal system, space group	Triclinic, *P* 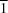
Temperature (K)	100
*a*, *b*, *c* ()	9.1350(18), 12.852(3), 13.607(3)
, , ()	103.69(3), 105.07(3), 101.25(3)
*V* (^3^)	1441.6(6)
*Z*	2
Radiation type	Synchrotron, = 0.62998
(mm^1^)	1.09
Crystal size (mm)	0.10 0.02 0.02

Data collection	
Diffractometer	ADSC Q210 CCD area detector
Absorption correction	Empirical (using intensity measurements) (*HKL3000sm* *SCALEPACK*; Otwinowski Minor, 1997 ▶)
*T*_min_, *T*_max_	0.899, 0.978
No. of measured, independent and observed [*I* > 2(*I*)] reflections	15474, 7929, 7758
*R*_int_	0.023
(sin /)_max_ (^1^)	0.696

Refinement	
*R*[*F* ^2^ > 2(*F* ^2^)], *wR*(*F* ^2^), *S*	0.037, 0.100, 1.03
No. of reflections	7929
No. of parameters	380
No. of restraints	3
H-atom treatment	H atoms treated by a mixture of independent and constrained refinement
_max_, _min_ (e ^3^)	0.74, 0.85

Computer programs: *PAL ADSC Quantum-210 ADX Software* (Arvai Nielsen, 1983 ▶), *HKL3000sm* (Otwinowski Minor, 1997 ▶), *SHELXS2014* and *SHELXL2014* (Sheldrick, 2008 ▶), *DIAMOND* (Brandenburg, 2007 ▶), *Mercury* (Macrae *et al.*, 2006 ▶). *PLATON* (Spek, 2009 ▶), *WinGX* (Farrugia, 2012 ▶) and *publCIF* (Westrip, 2010 ▶).

## supplementary crystallographic information

### Crystal data

**Table:**

[CrF_2_(C_5_H_5_N)_4_][ZnCl_3_(C_5_H_5_N)]·H_2_O	*Z* = 2
*M**_r_* = 675.23	*F*(000) = 686
Triclinic, *P*1	*D*_x_ = 1.556 Mg m^−^^3^
*a* = 9.1350 (18) Å	Synchrotron radiation, λ = 0.62998 Å
*b* = 12.852 (3) Å	Cell parameters from 70974 reflections
*c* = 13.607 (3) Å	θ = 0.4–33.6°
α = 103.69 (3)°	µ = 1.09 mm^−^^1^
β = 105.07 (3)°	*T* = 100 K
γ = 101.25 (3)°	Rod, purple
*V* = 1441.6 (6) Å^3^	0.10 × 0.02 × 0.02 mm

### Data collection

**Table:**

ADSC Q210 CCD area-detector diffractometer	7758 reflections with *I* > 2σ(*I*)
Radiation source: PLSII 2D bending magnet	*R*_int_ = 0.023
ω scans	θ_max_ = 26.0°, θ_min_ = 2.1°
Absorption correction: empirical (using intensity measurements) (*HKL3000sm**SCALEPACK*; Otwinowski & Minor, 1997)	*h* = −12→12
*T*_min_ = 0.899, *T*_max_ = 0.978	*k* = −17→17
15474 measured reflections	*l* = −18→18
7929 independent reflections	

### Refinement

**Table:**

Refinement on *F*^2^	3 restraints
Least-squares matrix: full	Hydrogen site location: mixed
*R*[*F*^2^ > 2σ(*F*^2^)] = 0.037	H atoms treated by a mixture of independent and constrained refinement
*wR*(*F*^2^) = 0.100	*w* = 1/[σ^2^(*F*_o_^2^) + (0.0457*P*)^2^ + 1.3104*P*] where *P* = (*F*_o_^2^ + 2*F*_c_^2^)/3
*S* = 1.03	(Δ/σ)_max_ = 0.001
7929 reflections	Δρ_max_ = 0.74 e Å^−^^3^
380 parameters	Δρ_min_ = −0.85 e Å^−^^3^

### Special details

**Table:**

Geometry. All e.s.d.'s (except the e.s.d. in the dihedral angle between two l.s. planes) are estimated using the full covariance matrix. The cell e.s.d.'s are taken into account individually in the estimation of e.s.d.'s in distances, angles and torsion angles; correlations between e.s.d.'s in cell parameters are only used when they are defined by crystal symmetry. An approximate (isotropic) treatment of cell e.s.d.'s is used for estimating e.s.d.'s involving l.s. planes.

### Fractional atomic coordinates and isotropic or equivalent isotropic displacement parameters (Å^2^)

**Table:**

	*x*	*y*	*z*	*U*_iso_*/*U*_eq_	Occ. (<1)
Cr1A	0.0000	0.5000	0.0000	0.01952 (8)	
F1A	0.20948 (11)	0.50546 (9)	0.01167 (8)	0.02511 (19)	
N1A	0.07291 (16)	0.61748 (11)	0.15115 (11)	0.0222 (2)	
N2A	−0.01489 (17)	0.36975 (11)	0.06719 (11)	0.0225 (3)	
C1A	0.2264 (2)	0.67086 (14)	0.20341 (14)	0.0270 (3)	
H1A	0.3020	0.6555	0.1696	0.032*	
C2A	0.2784 (2)	0.74746 (16)	0.30481 (15)	0.0319 (4)	
H2A	0.3875	0.7835	0.3395	0.038*	
C3A	0.1682 (2)	0.77060 (15)	0.35485 (15)	0.0317 (4)	
H3A	0.2009	0.8216	0.4246	0.038*	
C4A	0.0096 (2)	0.71743 (15)	0.30051 (15)	0.0308 (4)	
H4A	−0.0683	0.7325	0.3322	0.037*	
C5A	−0.0336 (2)	0.64231 (15)	0.19958 (15)	0.0267 (3)	
H5A	−0.1423	0.6067	0.1627	0.032*	
C6A	0.1138 (2)	0.34067 (16)	0.11190 (17)	0.0340 (4)	
H6A	0.2137	0.3811	0.1135	0.041*	
C7A	0.1058 (3)	0.25331 (17)	0.15599 (19)	0.0419 (5)	
H7A	0.1989	0.2343	0.1867	0.050*	
C8A	−0.0382 (3)	0.19486 (16)	0.15455 (16)	0.0369 (4)	
H8A	−0.0458	0.1355	0.1850	0.044*	
C9A	−0.1710 (3)	0.22358 (17)	0.10840 (19)	0.0382 (4)	
H9A	−0.2719	0.1841	0.1060	0.046*	
C10A	−0.1550 (2)	0.31122 (15)	0.06538 (17)	0.0308 (4)	
H10A	−0.2471	0.3306	0.0333	0.037*	
Cr2B	0.5000	0.5000	0.5000	0.02188 (8)	
F1B	0.47526 (13)	0.63979 (8)	0.49752 (9)	0.0287 (2)	
N1B	0.51583 (17)	0.46810 (12)	0.34563 (11)	0.0250 (3)	
N2B	0.25547 (17)	0.43358 (11)	0.44125 (11)	0.0233 (3)	
C1B	0.5327 (2)	0.55046 (16)	0.30013 (15)	0.0305 (3)	
H1B	0.5344	0.6229	0.3389	0.037*	
C2B	0.5478 (2)	0.5336 (2)	0.19893 (16)	0.0366 (4)	
H2B	0.5610	0.5936	0.1696	0.044*	
C3B	0.5431 (3)	0.4285 (2)	0.14199 (17)	0.0436 (5)	
H3B	0.5516	0.4147	0.0722	0.052*	
C4B	0.5259 (3)	0.3428 (2)	0.18750 (17)	0.0409 (5)	
H4B	0.5223	0.2697	0.1493	0.049*	
C5B	0.5140 (2)	0.36571 (16)	0.29025 (15)	0.0296 (3)	
H5B	0.5044	0.3074	0.3220	0.035*	
C6B	0.1601 (2)	0.49004 (14)	0.39770 (14)	0.0268 (3)	
H6B	0.2060	0.5583	0.3880	0.032*	
C7B	−0.0021 (2)	0.45198 (16)	0.36673 (15)	0.0311 (4)	
H7B	−0.0664	0.4930	0.3353	0.037*	
C8B	−0.0704 (2)	0.35292 (16)	0.38210 (15)	0.0314 (3)	
H8B	−0.1816	0.3259	0.3625	0.038*	
C9B	0.0272 (2)	0.29453 (15)	0.42650 (14)	0.0286 (3)	
H9B	−0.0163	0.2266	0.4377	0.034*	
C10B	0.1885 (2)	0.33643 (14)	0.45424 (13)	0.0261 (3)	
H10B	0.2549	0.2954	0.4835	0.031*	
Zn1C	0.70830 (2)	0.92169 (2)	0.25840 (2)	0.02919 (7)	0.631 (9)
Cl1C	0.7295 (8)	0.8517 (6)	0.4009 (5)	0.0297 (5)	0.631 (9)
Cl2C	0.6522 (2)	1.0811 (2)	0.2693 (4)	0.0462 (7)	0.631 (9)
Cl3C	0.5638 (4)	0.7835 (3)	0.10101 (11)	0.0511 (8)	0.631 (9)
Zn2C	0.70830 (2)	0.92169 (2)	0.25840 (2)	0.02919 (7)	0.369 (9)
Cl4C	0.5292 (3)	0.8210 (4)	0.10518 (17)	0.0433 (6)	0.369 (9)
Cl5C	0.6694 (3)	1.09905 (17)	0.3178 (5)	0.0361 (6)	0.369 (9)
Cl6C	0.7250 (16)	0.8618 (11)	0.3926 (10)	0.0352 (17)	0.369 (9)
N1C	0.93153 (18)	0.94763 (12)	0.24287 (12)	0.0262 (3)	
C1C	1.0575 (2)	1.00830 (14)	0.32876 (14)	0.0276 (3)	
H1C	1.0421	1.0336	0.3958	0.033*	
C2C	1.2089 (2)	1.03503 (17)	0.32268 (18)	0.0360 (4)	
H2C	1.2954	1.0788	0.3843	0.043*	
C3C	1.2318 (3)	0.9969 (2)	0.2255 (2)	0.0457 (5)	
H3C	1.3343	1.0140	0.2193	0.055*	
C4C	1.1034 (3)	0.9336 (2)	0.1378 (2)	0.0464 (5)	
H4C	1.1165	0.9061	0.0703	0.056*	
C5C	0.9552 (3)	0.91061 (17)	0.14915 (16)	0.0361 (4)	
H5C	0.8673	0.8671	0.0884	0.043*	
O1W	0.3884 (7)	0.0474 (5)	0.0419 (4)	0.162 (2)	
H1O1	0.462 (7)	0.063 (8)	0.102 (4)	0.243*	
H2O1	0.429 (10)	0.090 (7)	0.010 (6)	0.243*	

### Atomic displacement parameters (Å^2^)

**Table:**

	*U*^11^	*U*^22^	*U*^33^	*U*^12^	*U*^13^	*U*^23^
Cr1A	0.01680 (15)	0.02073 (16)	0.02493 (17)	0.00694 (12)	0.00807 (12)	0.01100 (13)
F1A	0.0185 (4)	0.0295 (5)	0.0311 (5)	0.0090 (4)	0.0093 (4)	0.0126 (4)
N1A	0.0219 (6)	0.0222 (6)	0.0271 (6)	0.0082 (5)	0.0095 (5)	0.0120 (5)
N2A	0.0243 (6)	0.0208 (6)	0.0243 (6)	0.0069 (5)	0.0080 (5)	0.0097 (5)
C1A	0.0248 (7)	0.0265 (7)	0.0303 (8)	0.0063 (6)	0.0093 (6)	0.0097 (6)
C2A	0.0319 (9)	0.0298 (8)	0.0311 (8)	0.0055 (7)	0.0078 (7)	0.0092 (7)
C3A	0.0411 (10)	0.0266 (8)	0.0298 (8)	0.0119 (7)	0.0122 (7)	0.0097 (6)
C4A	0.0378 (9)	0.0295 (8)	0.0341 (9)	0.0154 (7)	0.0181 (7)	0.0133 (7)
C5A	0.0266 (8)	0.0279 (8)	0.0322 (8)	0.0109 (6)	0.0136 (6)	0.0134 (6)
C6A	0.0260 (8)	0.0279 (8)	0.0444 (10)	0.0051 (6)	−0.0001 (7)	0.0185 (8)
C7A	0.0402 (11)	0.0300 (9)	0.0489 (12)	0.0064 (8)	−0.0034 (9)	0.0221 (9)
C8A	0.0562 (12)	0.0236 (8)	0.0344 (9)	0.0103 (8)	0.0147 (9)	0.0154 (7)
C9A	0.0481 (11)	0.0292 (9)	0.0552 (12)	0.0151 (8)	0.0344 (10)	0.0218 (9)
C10A	0.0311 (9)	0.0274 (8)	0.0450 (10)	0.0122 (7)	0.0214 (8)	0.0178 (7)
Cr2B	0.02634 (18)	0.01837 (16)	0.02080 (16)	0.00761 (13)	0.00498 (13)	0.00737 (12)
F1B	0.0342 (5)	0.0205 (4)	0.0322 (5)	0.0106 (4)	0.0076 (4)	0.0103 (4)
N1B	0.0231 (6)	0.0271 (7)	0.0233 (6)	0.0069 (5)	0.0048 (5)	0.0078 (5)
N2B	0.0278 (7)	0.0215 (6)	0.0202 (6)	0.0080 (5)	0.0055 (5)	0.0069 (5)
C1B	0.0281 (8)	0.0344 (9)	0.0282 (8)	0.0065 (7)	0.0053 (6)	0.0139 (7)
C2B	0.0253 (8)	0.0593 (13)	0.0329 (9)	0.0142 (8)	0.0112 (7)	0.0242 (9)
C3B	0.0354 (10)	0.0760 (16)	0.0312 (9)	0.0296 (11)	0.0167 (8)	0.0193 (10)
C4B	0.0379 (10)	0.0554 (13)	0.0323 (9)	0.0260 (10)	0.0134 (8)	0.0056 (9)
C5B	0.0251 (8)	0.0331 (8)	0.0292 (8)	0.0125 (7)	0.0068 (6)	0.0054 (7)
C6B	0.0323 (8)	0.0248 (7)	0.0255 (7)	0.0105 (6)	0.0080 (6)	0.0107 (6)
C7B	0.0324 (9)	0.0301 (8)	0.0333 (9)	0.0142 (7)	0.0078 (7)	0.0127 (7)
C8B	0.0285 (8)	0.0322 (9)	0.0331 (9)	0.0098 (7)	0.0082 (7)	0.0100 (7)
C9B	0.0320 (8)	0.0257 (7)	0.0274 (8)	0.0068 (6)	0.0076 (6)	0.0098 (6)
C10B	0.0311 (8)	0.0230 (7)	0.0231 (7)	0.0077 (6)	0.0053 (6)	0.0085 (6)
Zn1C	0.02382 (11)	0.02802 (11)	0.02942 (11)	−0.00267 (8)	−0.00014 (8)	0.01480 (8)
Cl1C	0.0301 (9)	0.0387 (8)	0.0288 (11)	0.0119 (8)	0.0107 (8)	0.0223 (11)
Cl2C	0.0305 (5)	0.0382 (8)	0.0857 (19)	0.0136 (5)	0.0246 (8)	0.0373 (11)
Cl3C	0.0450 (9)	0.0514 (12)	0.0299 (4)	−0.0202 (8)	−0.0006 (5)	0.0047 (5)
Zn2C	0.02382 (11)	0.02802 (11)	0.02942 (11)	−0.00267 (8)	−0.00014 (8)	0.01480 (8)
Cl4C	0.0341 (8)	0.0450 (13)	0.0311 (7)	0.0061 (9)	−0.0027 (6)	−0.0056 (7)
Cl5C	0.0309 (7)	0.0232 (6)	0.0530 (17)	0.0075 (5)	0.0144 (9)	0.0085 (8)
Cl6C	0.0364 (14)	0.052 (3)	0.0316 (16)	0.0196 (15)	0.0127 (10)	0.0318 (12)
N1C	0.0300 (7)	0.0211 (6)	0.0262 (7)	0.0052 (5)	0.0065 (5)	0.0089 (5)
C1C	0.0289 (8)	0.0231 (7)	0.0294 (8)	0.0080 (6)	0.0065 (6)	0.0079 (6)
C2C	0.0280 (9)	0.0355 (9)	0.0433 (10)	0.0103 (7)	0.0076 (8)	0.0130 (8)
C3C	0.0380 (11)	0.0567 (14)	0.0553 (13)	0.0235 (10)	0.0237 (10)	0.0222 (11)
C4C	0.0554 (14)	0.0520 (13)	0.0419 (11)	0.0256 (11)	0.0259 (10)	0.0124 (10)
C5C	0.0448 (11)	0.0312 (9)	0.0296 (9)	0.0108 (8)	0.0101 (8)	0.0061 (7)
O1W	0.193 (5)	0.152 (4)	0.114 (3)	−0.015 (3)	0.014 (3)	0.079 (3)

### Geometric parameters (Å, º)

**Table:**

Cr1A—F1A^i^	1.8645 (10)	C1B—H1B	0.9500
Cr1A—F1A	1.8645 (10)	C2B—C3B	1.375 (4)
Cr1A—N2A^i^	2.0873 (14)	C2B—H2B	0.9500
Cr1A—N2A	2.0873 (14)	C3B—C4B	1.388 (4)
Cr1A—N1A^i^	2.0926 (17)	C3B—H3B	0.9500
Cr1A—N1A	2.0926 (17)	C4B—C5B	1.396 (3)
N1A—C1A	1.348 (2)	C4B—H4B	0.9500
N1A—C5A	1.355 (2)	C5B—H5B	0.9500
N2A—C6A	1.341 (2)	C6B—C7B	1.381 (3)
N2A—C10A	1.343 (2)	C6B—H6B	0.9500
C1A—C2A	1.390 (3)	C7B—C8B	1.392 (3)
C1A—H1A	0.9500	C7B—H7B	0.9500
C2A—C3A	1.394 (3)	C8B—C9B	1.385 (3)
C2A—H2A	0.9500	C8B—H8B	0.9500
C3A—C4A	1.390 (3)	C9B—C10B	1.382 (3)
C3A—H3A	0.9500	C9B—H9B	0.9500
C4A—C5A	1.384 (3)	C10B—H10B	0.9500
C4A—H4A	0.9500	Zn1C—N1C	2.0752 (16)
C5A—H5A	0.9500	Zn1C—Cl2C	2.188 (2)
C6A—C7A	1.391 (3)	Zn1C—Cl3C	2.302 (2)
C6A—H6A	0.9500	Zn1C—Cl1C	2.303 (8)
C7A—C8A	1.374 (3)	Zn2C—N1C	2.0752 (16)
C7A—H7A	0.9500	Zn2C—Cl6C	2.126 (14)
C8A—C9A	1.375 (3)	Zn2C—Cl4C	2.196 (2)
C8A—H8A	0.9500	Zn2C—Cl5C	2.360 (2)
C9A—C10A	1.387 (3)	N1C—C5C	1.340 (2)
C9A—H9A	0.9500	N1C—C1C	1.348 (2)
C10A—H10A	0.9500	C1C—C2C	1.387 (3)
Cr2B—F1B	1.8609 (10)	C1C—H1C	0.9500
Cr2B—F1B^ii^	1.8609 (10)	C2C—C3C	1.381 (3)
Cr2B—N2B^ii^	2.0885 (17)	C2C—H2C	0.9500
Cr2B—N2B	2.0886 (17)	C3C—C4C	1.379 (4)
Cr2B—N1B	2.0916 (15)	C3C—H3C	0.9500
Cr2B—N1B^ii^	2.0916 (15)	C4C—C5C	1.385 (3)
N1B—C5B	1.347 (2)	C4C—H4C	0.9500
N1B—C1B	1.350 (2)	C5C—H5C	0.9500
N2B—C6B	1.349 (2)	O1W—H1O1	0.862 (10)
N2B—C10B	1.352 (2)	O1W—H2O1	0.855 (10)
C1B—C2B	1.389 (3)		
			
F1A^i^—Cr1A—F1A	180.0	C1B—N1B—Cr2B	120.77 (13)
F1A^i^—Cr1A—N2A^i^	90.11 (6)	C6B—N2B—C10B	118.27 (16)
F1A—Cr1A—N2A^i^	89.89 (6)	C6B—N2B—Cr2B	121.31 (12)
F1A^i^—Cr1A—N2A	89.89 (6)	C10B—N2B—Cr2B	120.20 (12)
F1A—Cr1A—N2A	90.11 (6)	N1B—C1B—C2B	122.68 (19)
N2A^i^—Cr1A—N2A	180.0	N1B—C1B—H1B	118.7
F1A^i^—Cr1A—N1A^i^	90.10 (6)	C2B—C1B—H1B	118.7
F1A—Cr1A—N1A^i^	89.90 (6)	C3B—C2B—C1B	118.8 (2)
N2A^i^—Cr1A—N1A^i^	91.02 (6)	C3B—C2B—H2B	120.6
N2A—Cr1A—N1A^i^	88.98 (6)	C1B—C2B—H2B	120.6
F1A^i^—Cr1A—N1A	89.90 (6)	C2B—C3B—C4B	119.32 (19)
F1A—Cr1A—N1A	90.10 (6)	C2B—C3B—H3B	120.3
N2A^i^—Cr1A—N1A	88.98 (6)	C4B—C3B—H3B	120.3
N2A—Cr1A—N1A	91.02 (6)	C3B—C4B—C5B	119.0 (2)
N1A^i^—Cr1A—N1A	180.0	C3B—C4B—H4B	120.5
C1A—N1A—C5A	117.88 (15)	C5B—C4B—H4B	120.5
C1A—N1A—Cr1A	121.38 (12)	N1B—C5B—C4B	121.85 (19)
C5A—N1A—Cr1A	120.73 (12)	N1B—C5B—H5B	119.1
C6A—N2A—C10A	117.91 (15)	C4B—C5B—H5B	119.1
C6A—N2A—Cr1A	121.44 (12)	N2B—C6B—C7B	122.20 (16)
C10A—N2A—Cr1A	120.64 (12)	N2B—C6B—H6B	118.9
N1A—C1A—C2A	122.59 (17)	C7B—C6B—H6B	118.9
N1A—C1A—H1A	118.7	C6B—C7B—C8B	119.28 (17)
C2A—C1A—H1A	118.7	C6B—C7B—H7B	120.4
C1A—C2A—C3A	119.05 (18)	C8B—C7B—H7B	120.4
C1A—C2A—H2A	120.5	C9B—C8B—C7B	118.69 (18)
C3A—C2A—H2A	120.5	C9B—C8B—H8B	120.7
C4A—C3A—C2A	118.61 (18)	C7B—C8B—H8B	120.7
C4A—C3A—H3A	120.7	C10B—C9B—C8B	119.09 (17)
C2A—C3A—H3A	120.7	C10B—C9B—H9B	120.5
C5A—C4A—C3A	119.13 (17)	C8B—C9B—H9B	120.5
C5A—C4A—H4A	120.4	N2B—C10B—C9B	122.45 (16)
C3A—C4A—H4A	120.4	N2B—C10B—H10B	118.8
N1A—C5A—C4A	122.72 (17)	C9B—C10B—H10B	118.8
N1A—C5A—H5A	118.6	N1C—Zn1C—Cl2C	105.20 (6)
C4A—C5A—H5A	118.6	N1C—Zn1C—Cl3C	100.81 (11)
N2A—C6A—C7A	122.21 (19)	Cl2C—Zn1C—Cl3C	114.17 (7)
N2A—C6A—H6A	118.9	N1C—Zn1C—Cl1C	104.3 (2)
C7A—C6A—H6A	118.9	Cl2C—Zn1C—Cl1C	119.5 (2)
C8A—C7A—C6A	119.26 (19)	Cl3C—Zn1C—Cl1C	110.34 (17)
C8A—C7A—H7A	120.4	N1C—Zn2C—Cl6C	105.5 (4)
C6A—C7A—H7A	120.4	N1C—Zn2C—Cl4C	110.44 (9)
C7A—C8A—C9A	119.03 (17)	Cl6C—Zn2C—Cl4C	118.5 (3)
C7A—C8A—H8A	120.5	N1C—Zn2C—Cl5C	106.82 (8)
C9A—C8A—H8A	120.5	Cl6C—Zn2C—Cl5C	103.3 (4)
C8A—C9A—C10A	118.84 (19)	Cl4C—Zn2C—Cl5C	111.42 (9)
C8A—C9A—H9A	120.6	C5C—N1C—C1C	118.27 (17)
C10A—C9A—H9A	120.6	C5C—N1C—Zn1C	122.44 (14)
N2A—C10A—C9A	122.74 (18)	C1C—N1C—Zn1C	119.22 (13)
N2A—C10A—H10A	118.6	C5C—N1C—Zn2C	122.44 (14)
C9A—C10A—H10A	118.6	C1C—N1C—Zn2C	119.22 (13)
F1B—Cr2B—F1B^ii^	180.0	N1C—C1C—C2C	122.33 (18)
F1B—Cr2B—N2B^ii^	90.15 (6)	N1C—C1C—H1C	118.8
F1B^ii^—Cr2B—N2B^ii^	89.85 (6)	C2C—C1C—H1C	118.8
F1B—Cr2B—N2B	89.85 (6)	C3C—C2C—C1C	118.9 (2)
F1B^ii^—Cr2B—N2B	90.15 (6)	C3C—C2C—H2C	120.6
N2B^ii^—Cr2B—N2B	180.00 (4)	C1C—C2C—H2C	120.6
F1B—Cr2B—N1B	90.00 (6)	C4C—C3C—C2C	118.9 (2)
F1B^ii^—Cr2B—N1B	90.01 (6)	C4C—C3C—H3C	120.5
N2B^ii^—Cr2B—N1B	88.16 (6)	C2C—C3C—H3C	120.5
N2B—Cr2B—N1B	91.84 (6)	C3C—C4C—C5C	119.3 (2)
F1B—Cr2B—N1B^ii^	90.00 (6)	C3C—C4C—H4C	120.4
F1B^ii^—Cr2B—N1B^ii^	90.00 (6)	C5C—C4C—H4C	120.4
N2B^ii^—Cr2B—N1B^ii^	91.84 (6)	N1C—C5C—C4C	122.3 (2)
N2B—Cr2B—N1B^ii^	88.16 (7)	N1C—C5C—H5C	118.9
N1B—Cr2B—N1B^ii^	180.0	C4C—C5C—H5C	118.9
C5B—N1B—C1B	118.29 (16)	H1O1—O1W—H2O1	104 (2)
C5B—N1B—Cr2B	120.92 (13)		
			
C5A—N1A—C1A—C2A	1.5 (2)	C1B—N1B—C5B—C4B	−1.3 (3)
Cr1A—N1A—C1A—C2A	−178.56 (13)	Cr2B—N1B—C5B—C4B	−179.40 (15)
N1A—C1A—C2A—C3A	−0.1 (3)	C3B—C4B—C5B—N1B	1.3 (3)
C1A—C2A—C3A—C4A	−1.1 (3)	C10B—N2B—C6B—C7B	0.2 (3)
C2A—C3A—C4A—C5A	1.0 (3)	Cr2B—N2B—C6B—C7B	−174.36 (13)
C1A—N1A—C5A—C4A	−1.6 (2)	N2B—C6B—C7B—C8B	0.9 (3)
Cr1A—N1A—C5A—C4A	178.42 (13)	C6B—C7B—C8B—C9B	−1.1 (3)
C3A—C4A—C5A—N1A	0.4 (3)	C7B—C8B—C9B—C10B	0.1 (3)
C10A—N2A—C6A—C7A	−0.3 (3)	C6B—N2B—C10B—C9B	−1.3 (2)
Cr1A—N2A—C6A—C7A	−179.28 (17)	Cr2B—N2B—C10B—C9B	173.40 (13)
N2A—C6A—C7A—C8A	−0.4 (4)	C8B—C9B—C10B—N2B	1.1 (3)
C6A—C7A—C8A—C9A	0.7 (3)	C5C—N1C—C1C—C2C	1.2 (3)
C7A—C8A—C9A—C10A	−0.4 (3)	Zn1C—N1C—C1C—C2C	−175.74 (14)
C6A—N2A—C10A—C9A	0.6 (3)	Zn2C—N1C—C1C—C2C	−175.74 (14)
Cr1A—N2A—C10A—C9A	179.61 (16)	N1C—C1C—C2C—C3C	−0.9 (3)
C8A—C9A—C10A—N2A	−0.2 (3)	C1C—C2C—C3C—C4C	0.0 (3)
C5B—N1B—C1B—C2B	0.2 (3)	C2C—C3C—C4C—C5C	0.4 (4)
Cr2B—N1B—C1B—C2B	178.31 (14)	C1C—N1C—C5C—C4C	−0.7 (3)
N1B—C1B—C2B—C3B	0.9 (3)	Zn1C—N1C—C5C—C4C	176.14 (17)
C1B—C2B—C3B—C4B	−0.9 (3)	Zn2C—N1C—C5C—C4C	176.14 (17)
C2B—C3B—C4B—C5B	−0.1 (3)	C3C—C4C—C5C—N1C	−0.1 (4)

Symmetry codes: (i) −*x*, −*y*+1, −*z*; (ii) −*x*+1, −*y*+1, −*z*+1.

### Hydrogen-bond geometry (Å, º)

**Table:**

*D*—H···*A*	*D*—H	H···*A*	*D*···*A*	*D*—H···*A*
O1*W*—H1*O*1···Cl2*C*^iii^	0.86 (1)	2.41 (2)	3.263 (6)	173 (8)
O1*W*—H2*O*1···Cl3*C*^iv^	0.86 (1)	2.48 (4)	3.281 (5)	157 (8)
O1*W*—H2*O*1···Cl4*C*^iv^	0.86 (1)	2.22 (2)	3.053 (6)	165 (8)
C2*B*—H2*B*···Cl3*C*	0.95	2.81	3.749 (4)	170
C3*B*—H3*B*···Cl3*C*^iv^	0.95	2.82	3.511 (3)	130
C3*C*—H3*C*···Cl2*C*^v^	0.95	2.71	3.627 (4)	162
C4*A*—H4*A*···Cl1*C*^vi^	0.95	2.82	3.717 (8)	158
C10*A*—H10*A*···Cl3*C*^i^	0.95	2.86	3.617 (4)	137
C10*B*—H10*B*···Cl1*C*^ii^	0.95	2.73	3.534 (9)	142

Symmetry codes: (i) −*x*, −*y*+1, −*z*; (ii) −*x*+1, −*y*+1, −*z*+1; (iii) *x*, *y*−1, *z*; (iv) −*x*+1, −*y*+1, −*z*; (v) *x*+1, *y*, *z*; (vi) *x*−1, *y*, *z*.
